# Three-dimensional printing model improves morphological understanding in acetabular fracture learning: A multicenter, randomized, controlled study

**DOI:** 10.1371/journal.pone.0191328

**Published:** 2018-01-17

**Authors:** Zhenfei Huang, Wenhao Song, Yaoshen Zhang, Qiang Zhang, Dongsheng Zhou, Xi Zhou, Yu He

**Affiliations:** 1 Department of Orthopaedics, Peking Union Medical College Hospital, Chinese Academy of Medical Sciences & Peking Union Medical College, Beijing, Beijing, China; 2 Department of Orthopaedics, Shandong Provincial Hospital Affiliated to Shandong University, Ji’nan, Shandong, China; 3 Department of Orthopaedics, Beijing Chaoyang Hospital, Capital Medical University, Beijing, Beijing, China; 4 Department of Orthopaedics, Beijing Ditan Hospital, Capital Medical University, Beijing, Beijing, China; Garvan Institute of Medical Research, AUSTRALIA

## Abstract

Conventional education results in unsatisfactory morphological understanding of acetabular fractures due to lack of three-dimensional (3D) details and tactile feedback of real fractures. Virtual reality (VR) and 3D printing (3DP) techniques are widely applied in teaching. The purpose of this study was to identify the effect of physical model (PM), VR and 3DP models in education of morphological understanding of acetabular fractures. 141 students were invited to participate in this study. Participants were equally and randomly assigned to the PM, VR and 3DP learning groups. Three-level objective tests were conducted to evaluate learning, including identifying anatomical landmarks, describing fracture lines, identifying classification, and inferring fracture mechanism. Four subjective questions were asked to evaluate the usability and value of instructional materials. Generally, the 3DP group showed a clear advantage over the PM and VR groups in objective tests, while there was no significant difference between the PM and VR groups. 3DP was considered to be the most valuable learning tool for understanding acetabular fractures. The findings demonstrate that 3DP modelling of real fractures is an effective learning instrument that can be used to understand the morphology of acetabular fractures and promote subjective interest.

## Introduction

A complete understanding of fracture morphology is often the first step in treating acetabular fractures. Accurate fracture classification is of great importance for surgeons, especially for beginners, to identify diagnosis, approach, reduction and fixation[1, 2]. However, conventional clinical education reportedly results in unsatisfactory understanding of fracture classification. The ideal education of fracture classification follows a process: from vision to touch, from plane to stereo, and from intact to fracture. Conventional education on fracture classification uses a combination of textbooks, radiographs, three-dimensional (3D) reconstruction images and intact physical models (PM); this method cannot provide information on 3D fracture morphology and tactile feedback of real fractures. Hence, there is a gap between education and real fractures, especially for complex fractures.

With advances in education techniques, several modalities have attempted to fill this gap through fracture morphological teaching. Training students in real surgery is considered the gold standard for learning fracture characteristics due to the use of multiple senses, direct interaction and tactile manipulation. However, the application is limited by a lack of appropriate case material, the possibility of increased surgical trauma and iatrogenic injury, and ethical dilemmas. Templating fracture lines onto a pelvic model was recommended by several authors[3, 4]; however, this method cannot enable direct observation of the spatial relationship between real fragments.

Virtual reality (VR) is widely applied in the teaching field and can be used to accurately reconstruct stereoscopic objects from digital data[5]. The tactile-based Virtual-Fracture-Carving Simulator was used to help teach trainees the complex acetabular spatial anatomy by drawing fracture lines on a virtual model[3]. Although observation using the simulator is still limited to the two-dimensional (2D) plane and there is a lack of real fractures, this interactive system was considered to promote better understanding of fracture anatomy. However, many studies have suggested that VR models have a controversial impact on education[6, 7].

The tactile feedback is considered to be an interface between imaging data and real fractures, which allows the students to feel resistance, contours, textures, and edges of fractures[8]. Promotion better understanding and retention of spatial information and relationships are the greatest advantages of the haptics. In fracture learning, tactile input is crucial to effectively master the complex structure of acetabulum and provide the basis for surgical reduction and fixation. Rapid prototyping, also known as 3D printing (3DP), has been used for preoperative planning, intraoperative assistance, and as an tactile feedback educational tool through direct creation of the patient-specific fracture[9–11]. Mashiko et al. developed a real elastic aneurysm model using 3DP for surgical simulation and training[12]. This method was considered useful for understanding complex aneurysm structures. Li et al. reported that the use of lifelike 3DP models significantly improved the anatomical teaching of spine fractures[13]; however, 3DP fracture models were not evaluated in comparison with intact models that were widely used in routine teaching. Furthermore, it remains unclear whether it was possible to use 3DP models to learn about acetabular fractures, which involve the most difficult morphology and classification.

Therefore, the purpose of this study was to identify the effect of using PM, VR and 3DP models for educating students in morphological understanding of acetabular fractures. The aim of this method was not to replace conventional education, but to bridge the gap between conventional education and true fractures. We hypothesized that using the 3DP models would significantly improve students’ anatomical knowledge and visuospatial understanding of acetabular fractures.

## Methods

### Study design

All experimental protocols were exempted by the Institutional Review Board of Peking Union Medical College Hospital (PUMCH). Authors obtained verbal informed consent from all subjects to participate this study.

This prospective, multicenter, randomized, controlled study was conducted at three sites across China, including PUMCH, Shandong Provincial Hospital Affiliated to Shandong University, and Beijing Ditan Hospital Affiliated to Capital Medical University from May 2016 to November 2016. Before formal study, a pilot experiment was conducted to determine the minimum sample size. At least seventeen participants were required in each group under our standard (minimum significant absolute difference in outcome score = 15%,α = 0.05, andβ = 0.2). Two-hundred-and-twelve students in the first year of three-year standardization resident training from these national training bases were invited to participate in this study. Students at this stage of study have basic knowledge of general anatomy and orthopedics, but no substantial knowledge of acetabular fractures. We explained to the trainees that the supplemental teaching in this study was independent of their course and the decision on whether to participate would not affect their grades on completion of the orthopedic course. All participants were required to perform an evaluation of medical background and visuospatial ability. One-hundred-and-forty-one students with similar criteria were equally assigned to either the PM, VR or 3DP learning groups using a randomization protocol. In PUMCH, the sex distribution was 50% females and 50% males to eliminate the suspected confounding factor of sex, whereas all participants were males in the other two centers.

### Learning groups

The learning process of each group was separated. There was an initial preparatory session for each group in which the same 60-minute lecture provided elementary knowledge of anatomy and Judet-Letournel classification of acetabular fractures. In this preparatory session, the anterior column, anterior wall, posterior column, posterior wall, iliopectineal line, ilioischial line, articular surface, weight-bearing dome, and quadrilateral plate of the acetabulum were displayed emphatically. Moreover, the spatial relationship between fracture lines and anatomical landmarks in each fracture type was introduced.

**Fig 1:**
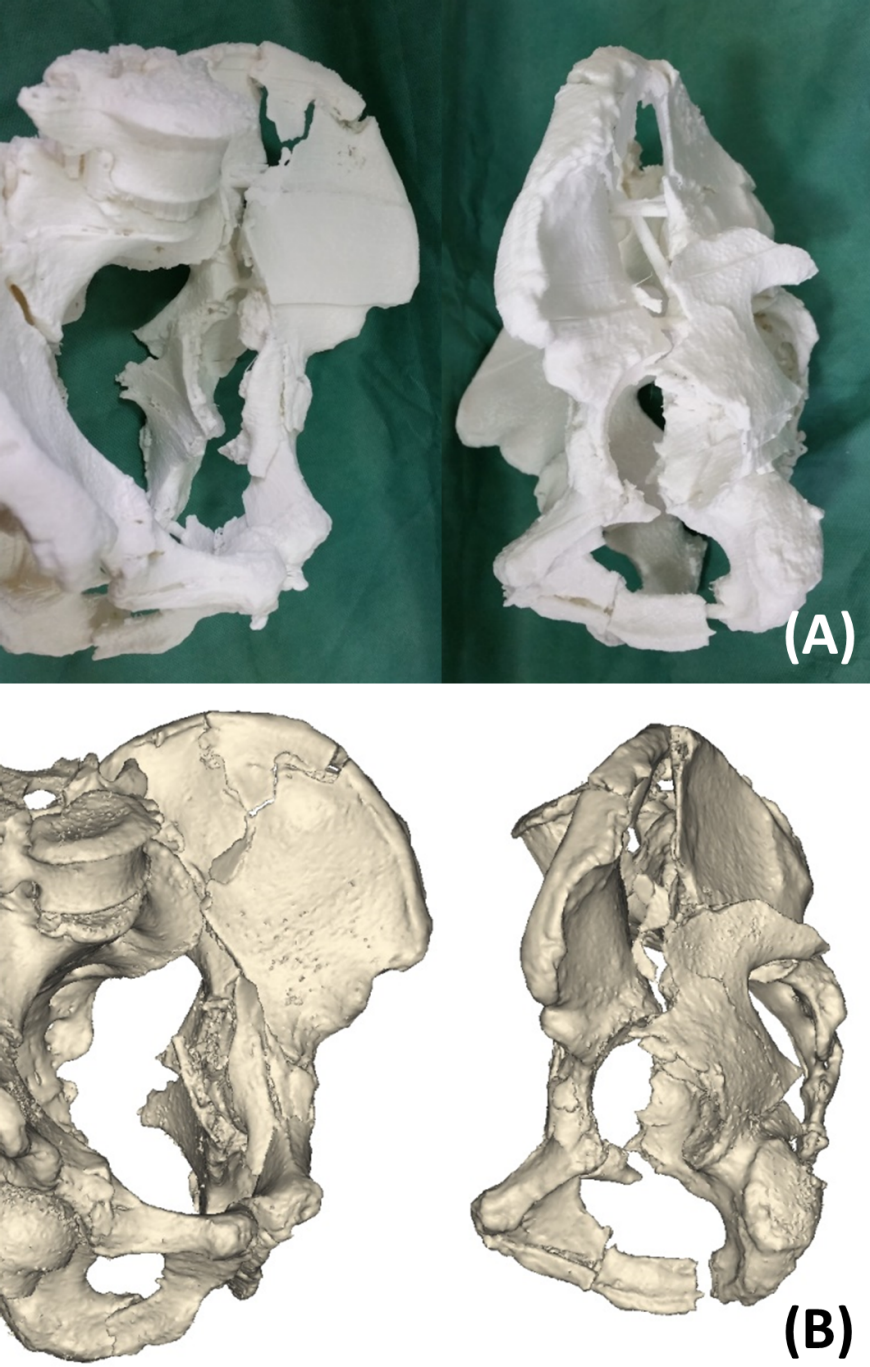
Example of three-dimensional printing (A) and virtual reality (B) models of acetabular factures.

After completing the initial learning phase, students were asked to review the information on acetabular fractures using a textbook, imaging data and each instrument for 30 minutes. In the PM group, students could touch the anatomical landmarks and the drawn fracture line on the physical pelvis model, just as in the conventional learning method. In addition to the conventional method, the VR group were given 3D digital models of acetabular fractures to aid in learning (Fig 1A). These models were created using a computed tomography (CT)-based technique with reconstruction software (3D-ORTHO; Waston Medical, Changzhou, China). VR models could be rotated and resized by clicking and dragging with a mouse. In addition to the conventional method, the 3DP group was given life-sized 3DP models of acetabular fractures to observe and touch (Fig 1B). The 3DP system (SRP400B; Waston Medical, Changzhou, China) used CT reconstruction data to build these models. In the manufacturing process, the free fragments were connected to the pelvis with connecting rods to prevent spatial relationship changes.

### Assessment

**Fig 2:**
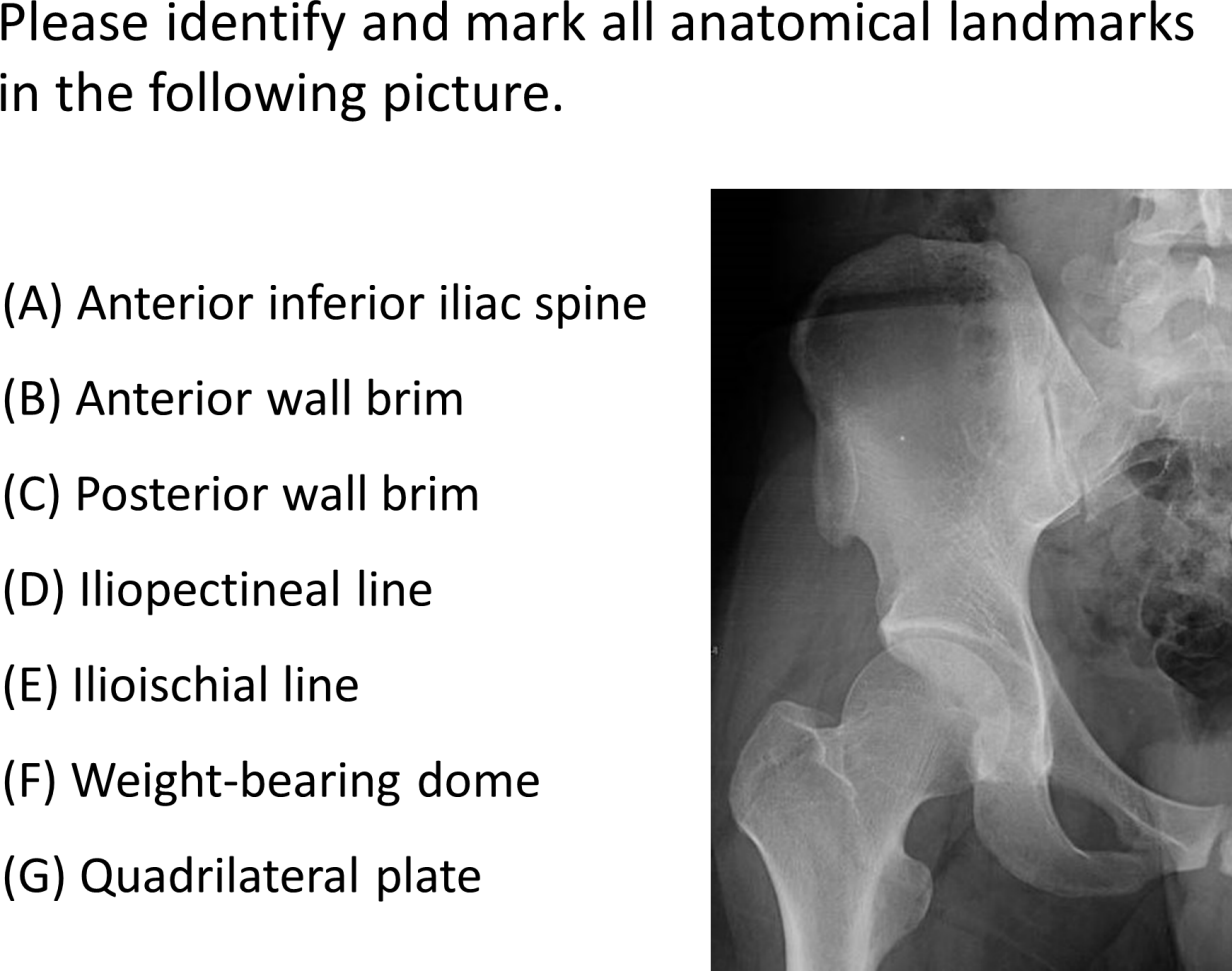
Example of questions used in the first level test.

**Fig 3:**
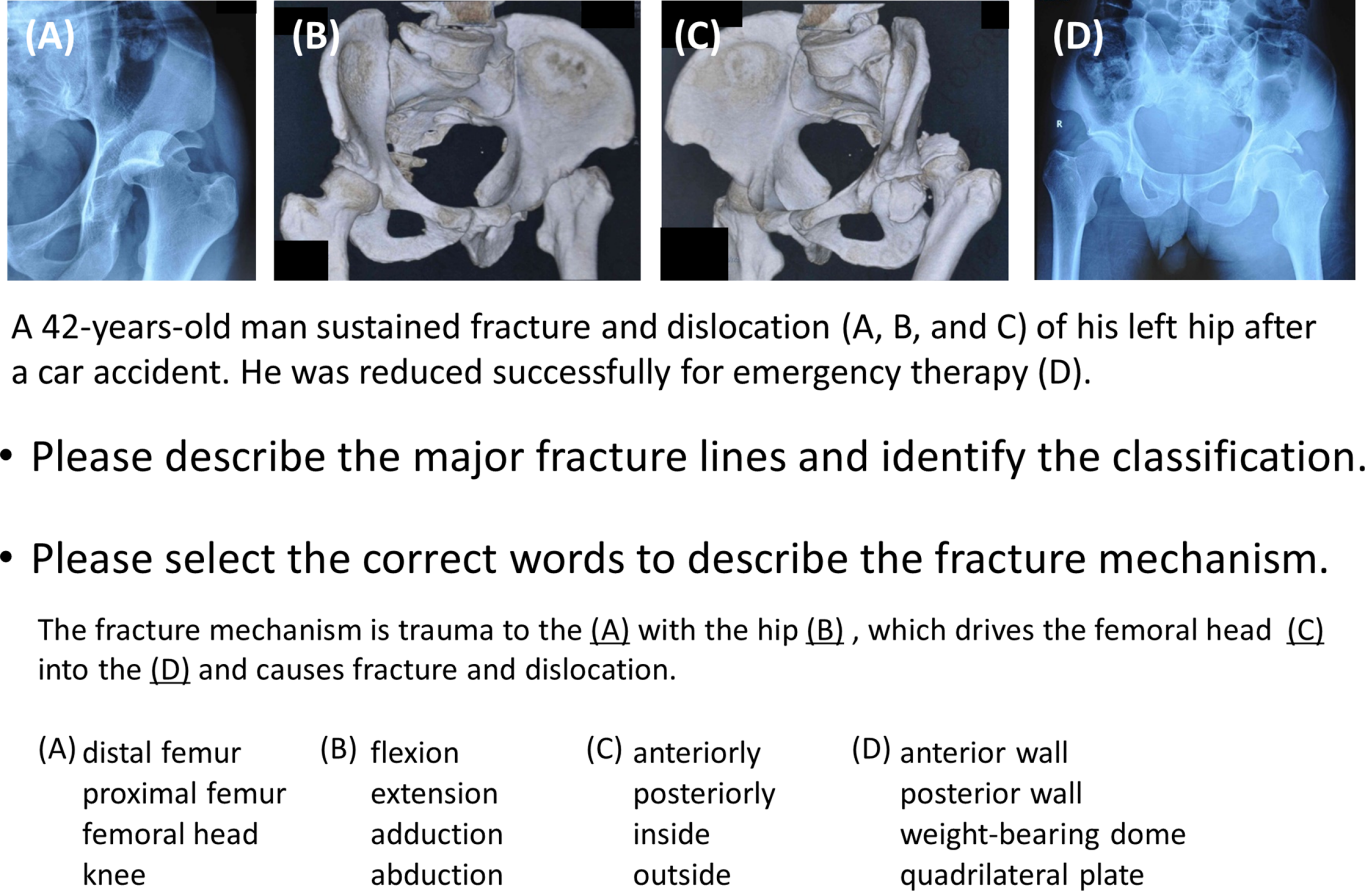
Example of questions used in the second and third level test.

Objective and subjective assessments were conducted after the learning process. The testing materials, such as imaging data, 3D digital models, and 3DP models, were different from the previous ones in initial learning phase. To evaluate the morphological understanding of acetabular fractures, three levels of testing were conducted. For the first level, participants were asked to identify seven anatomical landmarks (anterior inferior iliac spine, anterior wall brim, posterior wall brim, iliopectineal line, ilioischial line, weight-bearing dome, and quadrilateral plate) on normal pelvic radiographs (Fig 2). After collected the papers, the same seven landmarks were asked to identify on acetabular two-column fracture hemi-pelvic radiographs. For the second level, participants were asked to describe the direction of five major fracture lines and to identify the fracture classification on radiographs and 3D CT reconstruction of fractures (posterior wall, transverse with posterior wall and two-column fractures), which comprise the most common preoperative evaluation method (Fig 3A). For the third level, participants were asked to deduce the traumatic mechanism in three of the cases from the second level assessment (Fig 3B). This challenge question was intended to evaluate the value of the instructional materials. Posterior wall fractures, transverse with posterior wall fractures and two-column fractures were chosen as the fracture types for inclusion in testing because they account for approximately two-thirds of all acetabular fractures[4, 14, 15]. The complete Chinese and translated questionnaires are shown in S1 and S2 Files.

The three groups were required to fill out a feedback questionnaire regarding the learning instruments using a five-point Likert scale (strongly disagree, disagree, indifferent, agree, and strongly agree). The questionnaire asked four questions (Enjoyment, Ease of use, Good presentation of fractures, and Usefulness in learning fractures) under two topics (usability of learning materials and value in understanding fractures).

### Statistical analysis

Statistical analyses were performed with SPSS software (version 19.0; SPSS Inc, Chicago, IL). A Kolmogorov-Smirnov test was used to analyze normal distribution. An analysis of variance was performed to compare the means of all three groups. The results of the subjective questionnaires were evaluated with the Mann-Whitney U test. The level of statistical significance was defined as p < 0.05.

## Results

The medical background and visuospatial ability of the participants was similar among the three groups. The characteristic of participants was shown in Table 1. Sex-related differences were not observed in any of the assessments.

**Table 1:** The characteristic of participants.

		PM group	VR group	3DP group
**Sample size (n)**	PUMCH	20	20	20
	SPH	12	12	12
	BDH	15	15	15
**Age (years old)**		23.5±1.2	23.5±1.1	23.3±0.9
**Gender**	Female	10	10	10
	Male	37	37	37

The characteristic of participants.

3DP, Three-dimensional Printing; BDH, Beijing Ditan Hospital Affiliated to Capital Medical University; PUMCH, Peking Union Medical College Hospital; PM, Physical Model; SPH, Shandong Provincial Hospital Affiliated to Shandong University; VR

### Understanding of acetabular fractures

**Fig 4:**
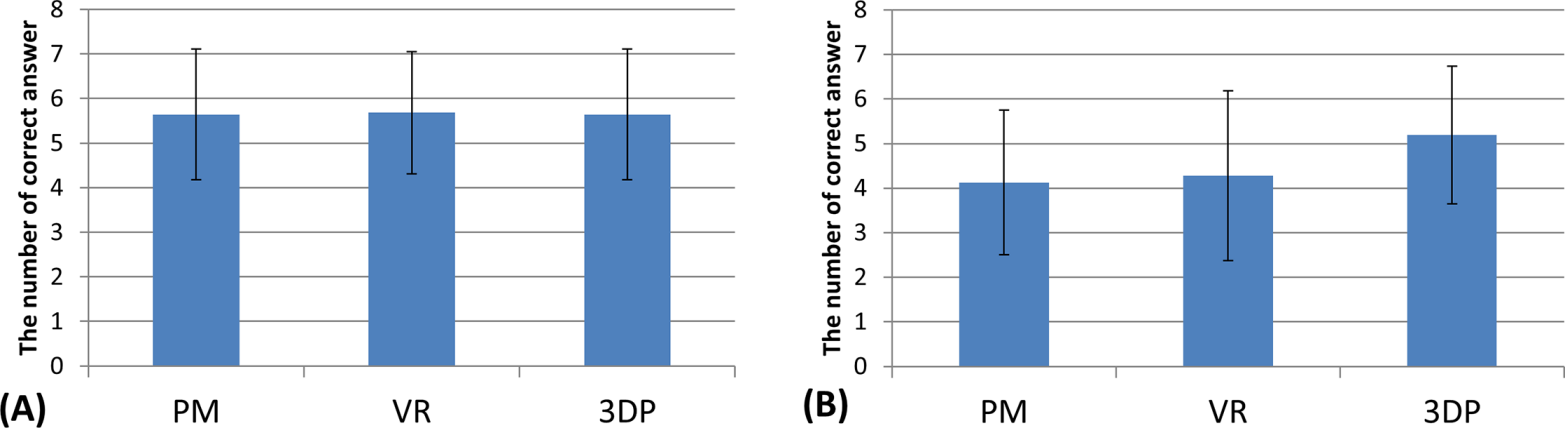
The results of recognizing anatomical landmarks in normal (A) and fracture (B) radiographs.

Regarding the identification of anatomical landmarks, VR and 3DP were considered to be better instructional materials compared with PM (Fig 4). In identifying landmarks on normal radiographs, there was no significant difference among the three groups. In identifying landmarks on fracture radiographs, the correct rate of the 3DP group was higher than that of the PM (p = 0.003) and VR groups (p = 0.010).

**Fig 5:**
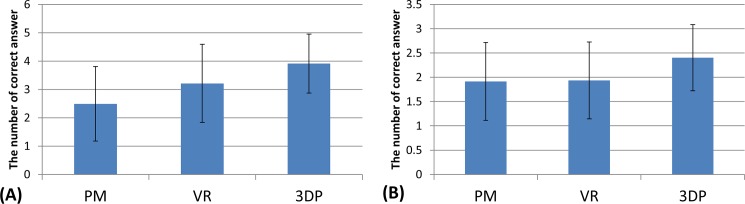
The results of identifying major fracture lines (A) and classification (B).

In testing of major fracture lines and classification, the 3DP group showed a clear advantage over the PM and VR groups (Fig 5). The rate of ‘all correct’ classifications was 27.7%, 27.7% and 51.1% in the PM, VR and 3DP groups, respectively. All students were able to accurately describe the direction of fracture lines and classification regarding acetabular posterior wall fracture. For description of major fracture lines, the correct rate of the VR group was higher than that of the PM group (p = 0.006); however, this advantage was not observed in the classification test (p = 0.892).

**Fig 6:**
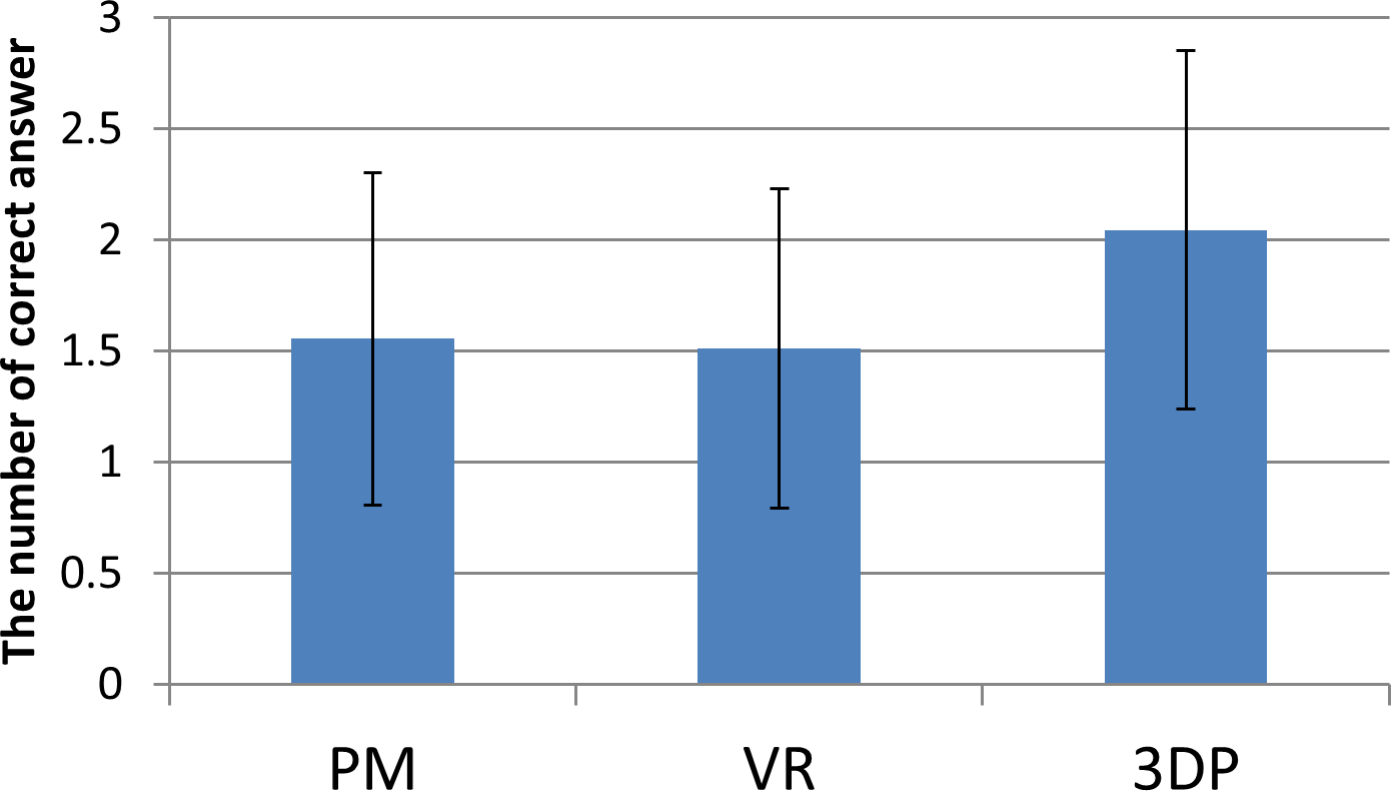
The results of inferring fracture mechanism.

In the challenge question, the proper deduction of traumatic mechanism proved difficult for the participants. The 3DP group had a higher reasoning capacity than the PM (p = 0.002) and VR groups (p = 0.003, Fig 6). Although all students in the 3DP group could infer that posterior impact of the femoral head on the acetabular wall was the mechanism of acetabular posterior fracture, only some participants were able to determine the injury position with the hip flexed.

### Subjective outcomes

**Fig 7:**
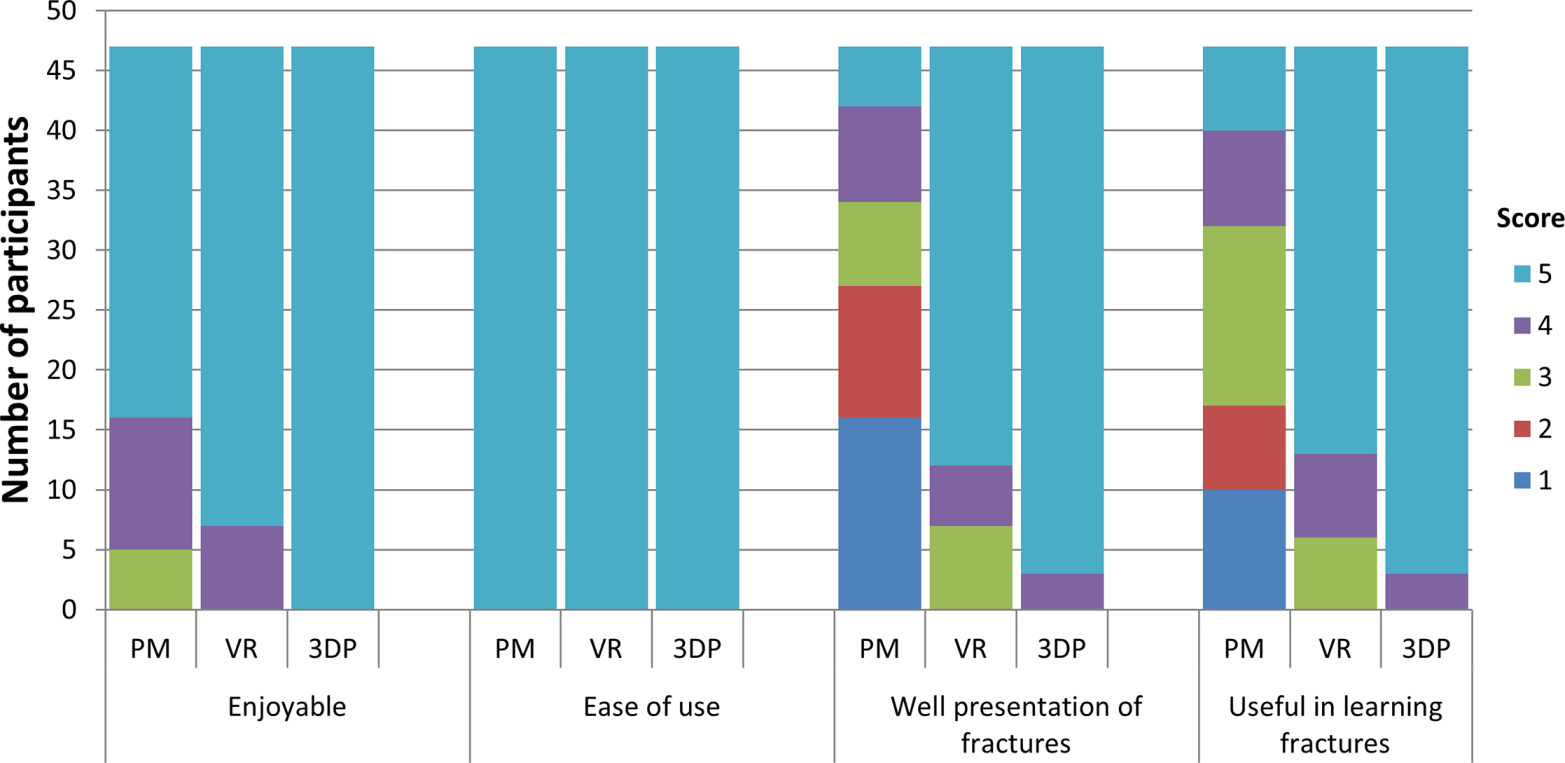
The results of feedback questionnaire.

The results of the feedback questionnaire are depicted in Fig 7. The usability of all learning materials was rated positively by all participants. All students could easily operate the tools, even those in the VR group. The VR and 3DP methods were more interesting for students than the PM method (p = 0.021 and p < 0.001). 3DP was considered to be the most valuable learning material for understanding acetabular fractures. The percentages of students responding to the statement “Good presentation of fractures” with “agree” or “strongly agree” were 25.5%, 85.1% and 100% in the PM, VR and 3DP groups, respectively. The percentages responding with “agree” or “strongly agree” to the statement “Usefulness in learning fractures” in the PM, VR and 3DP groups were 31.9%, 87.2% and 100%, respectively.

## Discussion

The present findings demonstrate that 3DP models of real fractures were effective tools for teaching the morphology of acetabular fractures. The ultimate goal is to use 3DP models as a complementary aid to conventional education methods, as opposed to a replacement. The advantage of 3DP models is the ability to enhance the understanding of the spatial relationship between anatomical landmarks and fracture lines, not only from 3D fracture morphology, but also from tactile feedback of real fractures.

According to Pahuta et al., there are four key points to fracture anatomy: identifying landmarks by vision, identifying landmarks by touch, understanding the relationship of fractures, and converting the spatial relationship into the patient’s position[3]. Conventional education of acetabular fractures often involves a combination of textbooks and intact pelvic models, which can provide fracture morphology in 2D vision and tactile feedback of the non-fracture condition. There is a gap between education and real fractures, especially in learning complex structures. The information provided by 3D fracture morphology and haptic feedback of real fractures could bridge this gap. The 3DP technique, which accurately reconstructs stereoscopic objects from CT scans, is a promising method to achieve this.

In our opinion, the ideal education of anatomy follows a process: from vision to touch, from plane to stereo, and from intact to fracture. With advances in education, several modalities have attempted to achieve this goal. A tactile-based Virtual-Fracture-Carving Simulator was used to help teach trainees the spatial anatomy of the acetabular complex by drawing fracture lines on virtual models[3]. This interactive system was believed to promote better understanding of fracture anatomy, but observation was still limited to 2D images and there was a lack of real fractures. Preece et al. evaluated the effect of 3DP models in teaching the complex spatial relationships of the equine foot, and suggested that 3DP models have a significant advantage over textbooks and VR by improving visuospatial understanding[16]; however, this previous study explored the teaching of physiological rather than pathological anatomy. Li et al. reported that lifelike 3DP models significantly improved the teaching of the anatomy of spine fractures[13]; however, 3DP fracture models were not evaluated by systematic comparison with physical models. Furthermore, it remains unclear whether it was possible to use 3DP models to learn about acetabular fractures, which involve the most difficult morphology and classification.

In the present study, we made vivid models of acetabular fractures to help students learn morphology. It is clear that 3D visual and tactile information of true fractures could only be offered by the 3DP models, not by the PM and VR models. By touching the anatomical landmarks and fracture lines in the 3DP models, students could obtain spatial details of fracture morphology that could not be provided by the other methods. This may explain why the 3DP group showed a clear advantage over the PM and VR groups in identifying landmarks, describing major fracture lines and classifying fractures. Interestingly, all participants were able to accurately determine simple fracture lines and classification regarding posterior wall fracture. However, most students in the PM and VR groups failed to correctly identify complex fracture lines and classification involving transverse with posterior wall fractures and two-column fractures. The present results indicate that haptic information may be unnecessary for understanding simple spatial relationships, but crucial to learning about complicated structures like complex acetabular fractures. This could also explain why there was no significant difference among the three groups in identifying landmarks on normal radiographs.

Participants were asked to deduce the traumatic mechanism of three common acetabular fractures, which was intended to assess the value of the instructional materials. The inference process required the integration of all kinds of information from existing to new knowledge. The higher correct rate obtained by participants using the 3DP models may be attributable to the ability to integrate information to form a complete chain from vision to touch, from plane to stereo, and from intact to fracture. The cognitive transfer from intact to broken acetabulum could be facilitated using physical and 3DP models. The hand-held interactive experience enhanced critical thinking about the fracture mechanism, which may be why only some participants in the 3DP group were able to determine the injury position in the flexed hip in the process of acetabular posterior fracture.

In the subjective tests, 3DP was considered to be the most valuable and enjoyable learning instrument. There is no doubt that advanced technology and novel methods are more attractive to students. This attraction can be transformed into a positive attitude toward the education resource. Due to inherent defects, the PM models received the lowest score in “Good presentation of fractures” and “Usefulness in learning fractures” without vivid fracture presentation. The 3DP model was intended to remedy this defect. Students had a positive response toward the usability of learning materials, even in the VR group; this is in agreement with previous studies that reported that the VR technique is well received by trainees[17, 18].

It is controversial as to whether VR can increase spatial understanding of anatomy. Evidence has illustrated that a positive effect was observed in assimilating anatomical structures and spatial relationships[3, 19, 20]. However, some scholars reported that PM could be more helpful than VR[21, 22]. From our objective results, a better understanding of acetabular fractures was not observed in the VR group compared with the PM group. Although stereoscopic sensation was provided by VR, the learning process was still limited to 2D pictures on a computer screen, which may require more cognitive capability. In the subjective tests, VR was considered to be a more valuable and enjoyable learning method than PM. This creates questions regarding the real validity of the VR technique in teaching and learning. The subjective advantage may transform and be reflected in the long-term learning process. However, larger samples and long-term follow-up are needed to provide conclusive evidence.

It is generally accepted that there are sex-related differences in visuospatial ability[23, 24]. However, this difference was not observed in our results. Because of the limited number of female participants in this study, it is difficult to make a definite qualitative conclusion.

There were limitations to the current study. The first limitation is that long-term retention of knowledge was not tested. However, the primary aim of the current study was improvement of understanding, not improvement of memorizing, spatial relationships in acetabular fractures. Logically, better understanding and a positive attitude are valuable for long-term retention of information. The second limitation is that all participants were volunteers. In this situation, the subjects who were less motivated and may have scored lower on the tests were not excluded. However, this bias would have been minimized by the randomization protocol used to assign students to groups. With the advancement of technology, there will be more advanced equipment for medical education, as you mentioned. The third limitation is that some 3D VR systems[25], providing better learning experience, were not included in the present study because this kind of device has not been used in our routine education. Whether 3D VR systems were more effective should be investigated in further study. One thing should be kept in mind that education significance is just as important as statistical significance. In present study, statistical significance just prompted us that 3D printing models of real fractures is an effective tool to understand the morphology of acetabular fractures and promote subjective interest possibly. From pedagogical perspectives, this model is a promising tool to clinic education. However, whether the 3DP model is effective remains to be evaluated in long-term real teaching process instead of one experiment.

In conclusion, 3DP models of real fractures were effective learning tools to promote morphological understanding and subjective interest in acetabular fractures. This advantage is attributed to not only presentation of 3D fracture morphology, but also tactile feedback of real fractures. The ultimate goal is for 3DP models to be used as a valid and complementary aid to conventional education methods, as opposed to a replacement.

## Supporting information

S1 File The complete Chinese questionnaires. (PDF)

S2 File The complete English questionnaires. (PDF)
