# Supplementary material for: Three-dimensional printing model improves morphological understanding in acetabular fracture learning: A multicenter, randomized, controlled study
Source: PLoS One. 2018 Jan 17;13(1):e0191328. doi: 10.1371/journal.pone.0191328 (PMC5771611; doi:10.1371/journal.pone.0191328)
Supplement: S1 File — (PDF) [file pone.0191328.s001.pdf]

试卷编码：51

# 髋臼骨折学习调查问卷（一）

年龄：________ 性别：________

一、 请在图片中标出以下解剖标志

（1）髂前下棘

（2）髋臼前壁缘

（3）髋臼后壁缘

（4）髂耻线

（5）髂坐线

（6）髋臼负重顶

（7）髋臼四边体

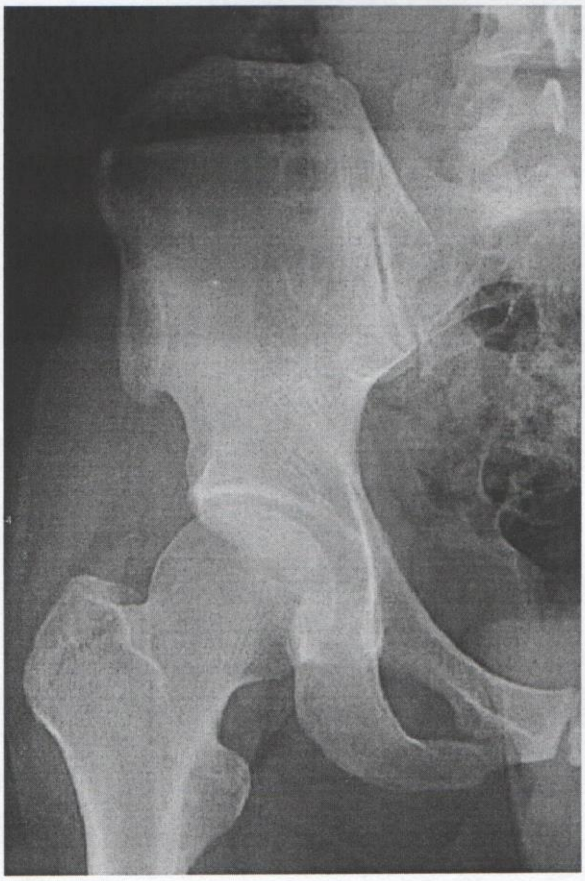

（不清楚见幻灯片）

# 髋臼骨折学习调查问卷（二）

一、 请在图片中标以下解剖标志

（1）髂前下棘
（2）髋臼前壁缘
（3）髋臼后壁缘
（4）髂耻线
（5）髂坐线
（6）髋臼负重顶
（7）髋臼四边体

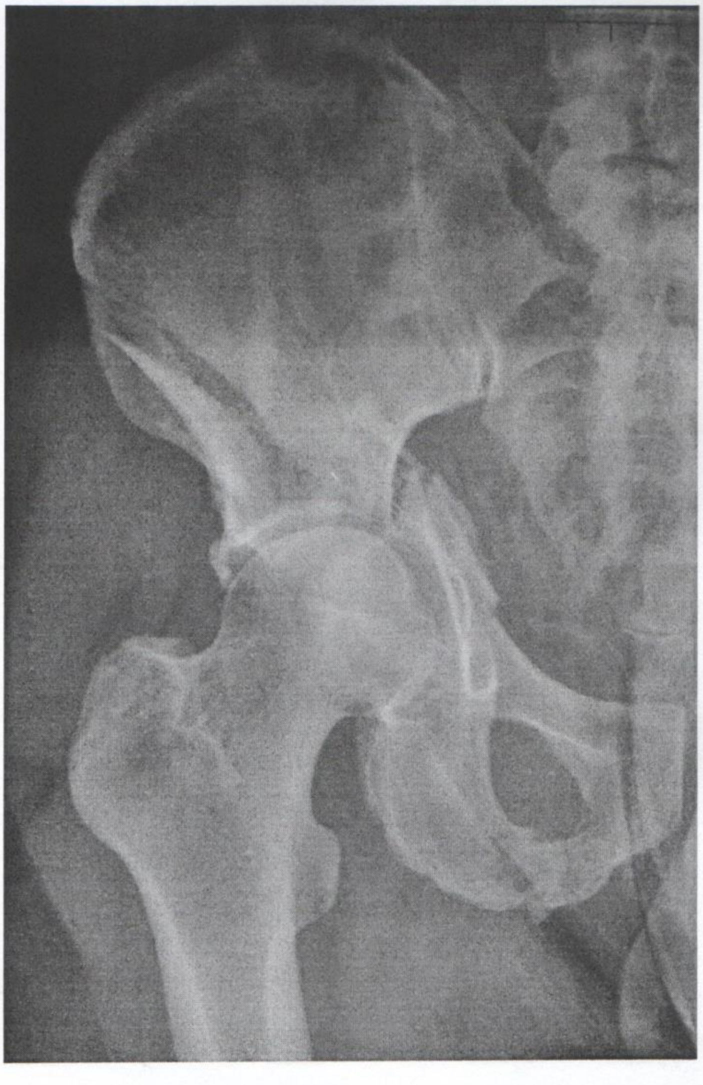

（不清楚见幻灯片）

# 髋臼骨折学习调查问卷（三）

一、　请依据病历及影像学资料描述该髋臼骨折的骨折线走行。

该患者为 42 岁男性，因车祸伤入院，发现左侧髋臼骨折并脱位，予以急诊复位。

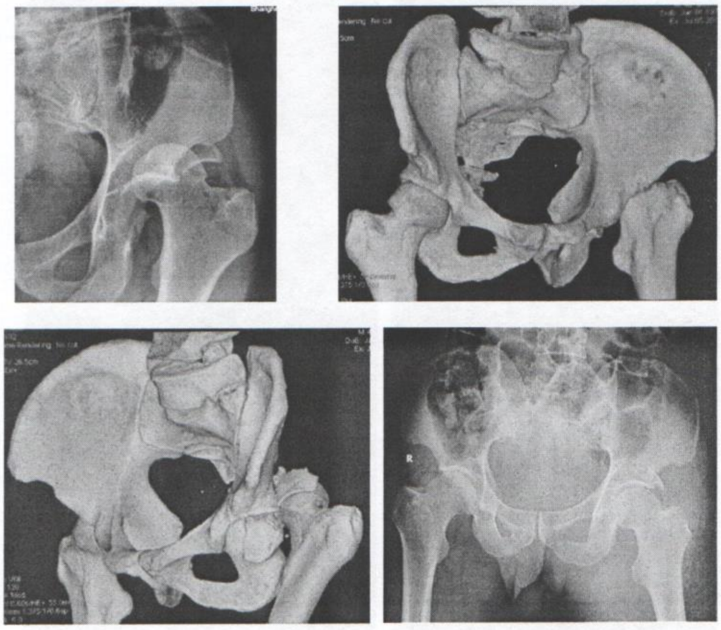

（不清楚见幻灯片）

二、　请对该髋臼骨折进行分型（Judet-Letournel 分型）

Judet-Letournel 分型：
前壁骨折、后壁骨折、前柱骨折、后柱骨折、横行骨折
横行伴后壁骨折、前方伴后半横形骨折、后壁伴后柱骨折、T 形骨折、双柱骨折

三、　请推测该髋臼骨折的致伤机制，将正确的词语进行填空

该髋臼骨折及脱臼机制为：当髋关节处于<u>（1）</u>位，外力作用于<u>（2）</u>，使股骨头<u>（3）</u>撞击<u>（4）</u>所致。

（1）A. 伸直
B. 屈曲
C. 内收
D. 外展

（2）A. 股骨远端
B. 股骨近端
C. 股骨头
D. 膝关节

（3）A. 向前
B. 向后
C. 向内
D. 向外

（4）A. 前壁
B. 后壁
C. 负重顶
D. 四边体

试卷编码：51

# 髋臼骨折学习调查问卷（四）

## 一、 请依据病历及影像学资料描述该髋臼骨折的骨折线走行。

该患者为 37 岁男性，因车祸伤导致左侧髋臼骨折。

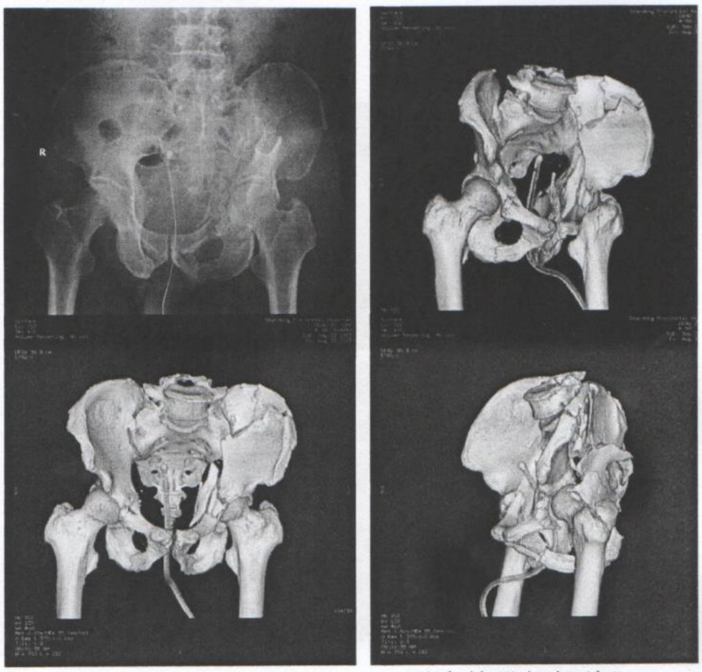

（不清楚见幻灯片）

## 二、 请对该髋臼骨折进行分型（Judet-Letournel 分型）

> Judet-Letournel 分型：
> 前壁骨折、后壁骨折、前柱骨折、后柱骨折、横行骨折
> 横行伴后壁骨折、前方伴后半横形骨折、后壁伴后柱骨折、T 形骨折、双柱骨折

## 三、 请推测该髋臼骨折的致伤机制，将正确的词语进行填空

该髋臼骨折为<u>（1）</u>暴力作用于髋臼的<u>（2）</u>损伤所致，<u>（3）</u>暴力自<u>（4）</u>作用于髋臼，使髋臼承重部分与髂骨翼分离。

（1）A. 直接
B. 间接

（2）A. 高能量
B. 低能量

（3）A. 垂直
B. 侧方
C. 旋转
D. 前后

（4）A. 股骨
B. 骶骨
C. 大转子
D. 小转子

# 髋臼骨折学习调查问卷（五）

一、　请依据病历及影像学资料描述该髋臼骨折的骨折线走行。

该患者为 46 岁男性，因坠落伤导致左侧髋臼骨折。

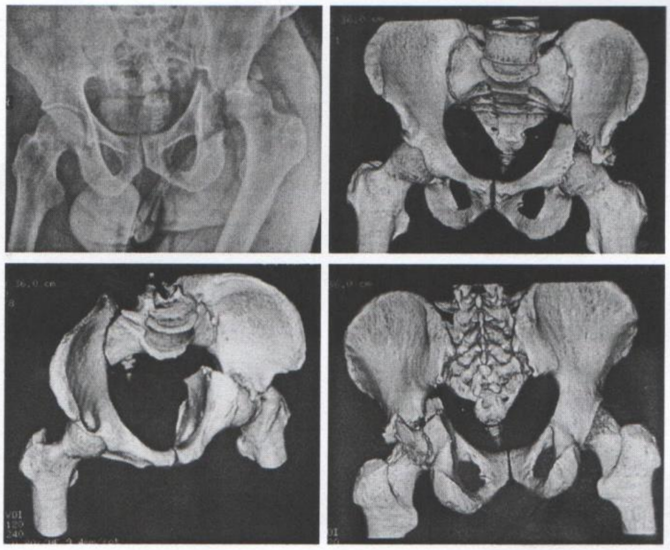

（不清楚见幻灯片）

二、　请对该髋臼骨折进行分型（Judet-Letournel 分型）

Judet-Letournel 分型：
前壁骨折、后壁骨折、前柱骨折、后柱骨折、横行骨折
横行伴后壁骨折、前方伴后半横形骨折、后壁伴后柱骨折、T 形骨折、双柱骨折

三、　请推测该髋臼骨折的致伤机制，将正确的词语进行填空

该髋臼骨折为直接暴力作用于<u>**（1）**</u>所致，此时髋关节处于屈曲<u>**（2）**</u>、外展<u>**（3）**</u>位。

| | | |
|---|---|---|
| （1）A. 屈曲位膝关节 | （2）A. 0° | （3）A. 0° |
| B. 腹部 | B. 15° | B. 10° |
| C. 腰骶部 | C. 45° | C. 25° |
| D. 大转子 | D. 90° | D. 60° |

# 髋臼骨折学习调查问卷（六）

一、 请选择您对以下描述的看法

（1） 你很享受这个学习过程。

| 非常同意 | 同意 | 无意见 | 不同意 | 非常不同意 |
|---|---|---|---|---|

（2） 你认为这种学习工具简单易用。

| 非常同意 | 同意 | 无意见 | 不同意 | 非常不同意 |
|---|---|---|---|---|

（3） 你认为这种学习工具可以很好的展现出髋臼骨折的形态。

| 非常同意 | 同意 | 无意见 | 不同意 | 非常不同意 |
|---|---|---|---|---|

（4） 你认为这种学习工具有助于学习髋臼骨折。

| 非常同意 | 同意 | 无意见 | 不同意 | 非常不同意 |
|---|---|---|---|---|
