# Supplementary material for: Three-dimensional printing model improves morphological understanding in acetabular fracture learning: A multicenter, randomized, controlled study
Source: PLoS One. 2018 Jan 17;13(1):e0191328. doi: 10.1371/journal.pone.0191328 (PMC5771611; doi:10.1371/journal.pone.0191328)
Supplement: S2 File — (PDF) [file pone.0191328.s002.pdf]

Acetabular fracture Learning Questionnaire(1)

Gender:_______ Age:_______

1. Please determine the following anatomical landmarks in the radiograph.

(1) Anterior inferior iliac spine
(2) Anterior wall brim
(3) Posterior wall brim
(4) Iliopectineal line
(5) Ilioischial line
(6) Weight-bearing dome
(7) Quadrilateral plate

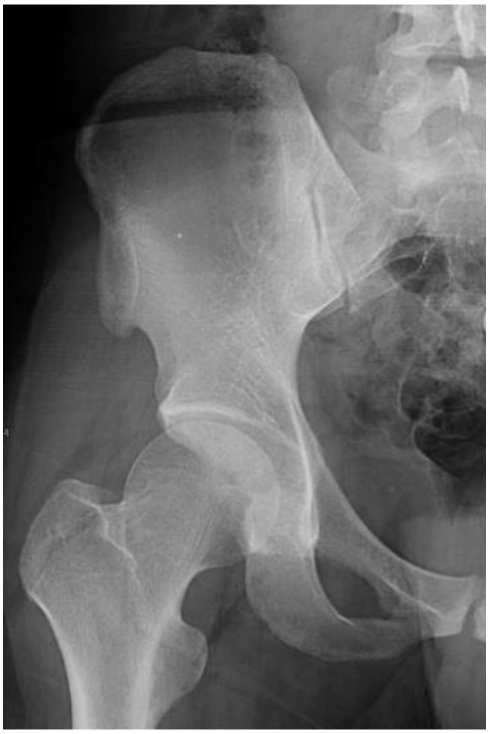

(Please see the slide, if the picture is not clear.)

Acetabular fracture Learning Questionnaire(2)

1. Please determine the following anatomical landmarks in the radiograph.

(1) Anterior inferior iliac spine

(2) Anterior wall brim

(3) Posterior wall brim

(4) Iliopectineal line

(5) Ilioischial line

(6) Weight-bearing dome

(7) Quadrilateral plate

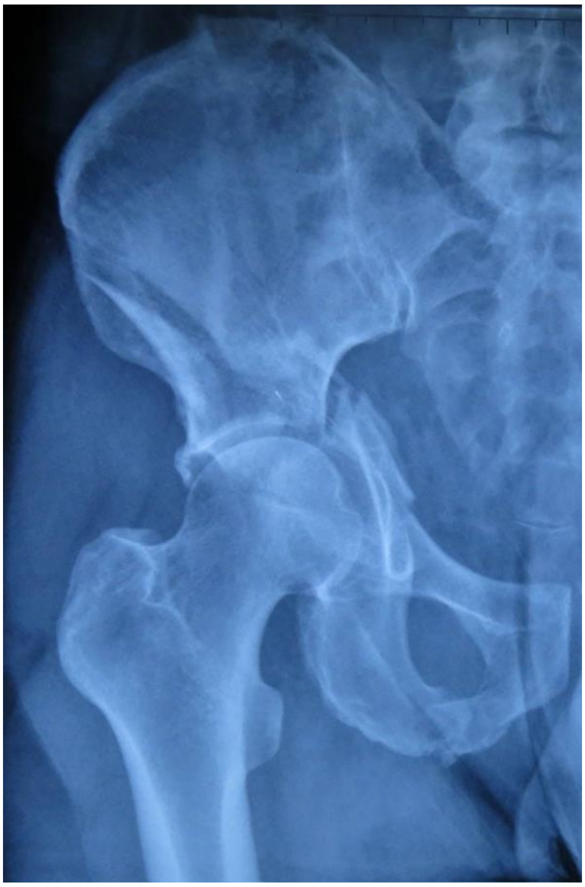

(Please see the slide, if the picture is not clear.)

Questionnaire ID:

Acetabular fracture Learning Questionnaire(3)

1. Please describe the fracture line of this acetabular fracture according to the medical record and imaging data.

The patient (42/M) was admitted to the hospital due to a car accident. Left acetabular fracture with dislocation was found. He was reduced successfully for emergency therapy.

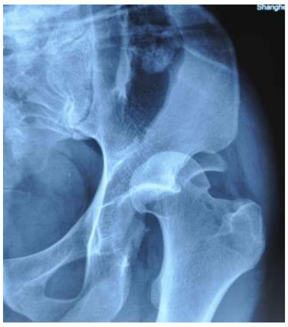

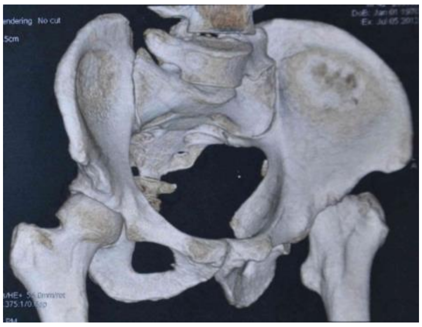

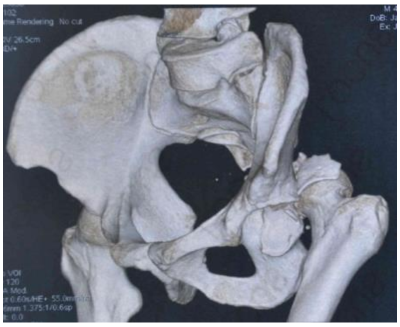

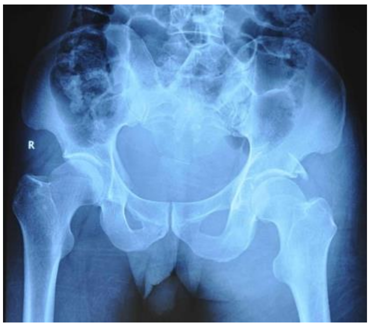

(Please see the slide, if the picture is not clear.)

2. Please classify this fracture according to Judet-Letournel classification.

> Judet-Letournel classification：
> Anterior wall, posterior wall, anterior column, posterior column, transverse, transverse with posterior wall, T-shaped, associated both-column, anterior column or wall with posterior hemitransverse, and posterior column with posterior wall

3. Please choose the right words to fill in the blanks to infer the mechanism of this fracture.

The fracture mechanism is trauma to the **(1)** with the hip **(2)**, which drives the femoral head **(3)** into the **(4)** and causes fracture and dislocation

(1)	(2)	(3)	(4)
A. distal femur	A. flexion	A. anteriorly	A. anterior wall
B. proximal femur	B. extension	B. posteriorly	B. posterior wall
C. femoral head	C. adduction	C. inside	C. weight-bearing dome
D. knee	D. abduction	D. outside	D. quadrilateral plate

Acetabular fracture Learning Questionnaire(4)

1. Please describe the fracture line of this acetabular fracture according to the medical record and imaging data.

The patient (37/M) had left acetabular fractures due to car accident.

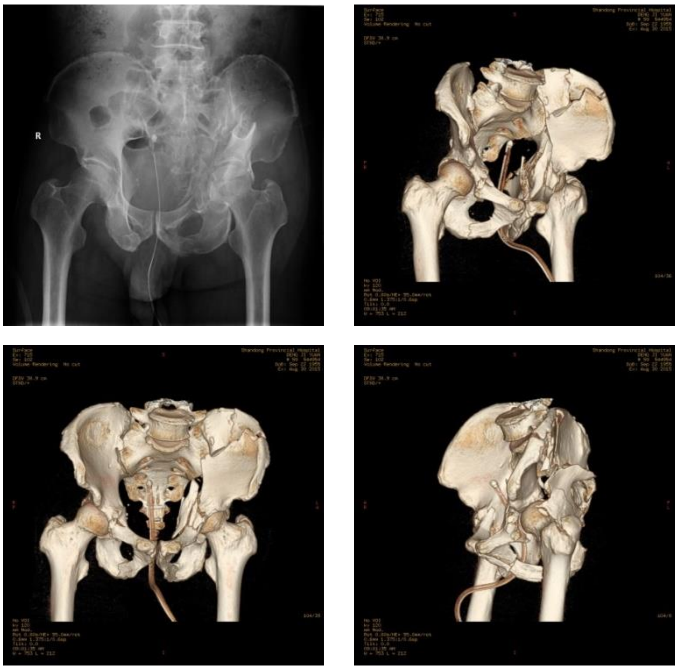

(Please see the slide, if the picture is not clear.)

2. Please classify this fracture according to Judet-Letournel classification.

> Judet-Letournel classification：
> Anterior wall, posterior wall, anterior column, posterior column, transverse, transverse with posterior wall, T-shaped, associated both-column, anterior column or wall with posterior hemitransverse, and posterior column with posterior wall

3. Please choose the right words to fill in the blanks to infer the mechanism of this fracture.

The acetabular fracture is caused by **(1)** injury **(2)** to the acetabulum, and **(3)** violence acts on the acetabulum from **(4)**, separating the weight-bearing portion part from the Iliac wing.

(1)	(2)	(3)	(4)
A. high-energy	A. directly	A. vertical	A. femur
B. low-energy	B. indirectly	B. lateral	B. sacrum
		C. rotated	C. greater trochanter
		D. anteroposterior	D. lesser trochanter

Acetabular fracture Learning Questionnaire(5)

1. Please describe the fracture line of this acetabular fracture according to the medical record and imaging data.

The patient (46/M) suffered left acetabular fractures due to falling injury.

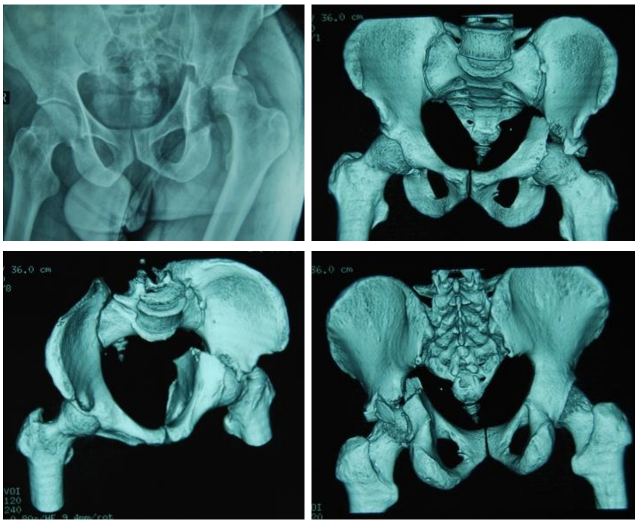

(Please see the slide, if the picture is not clear.)

2. Please classify this fracture according to Judet-Letournel classification.

> Judet-Letournel classification：
> Anterior wall, posterior wall, anterior column, posterior column, transverse, transverse with posterior wall, T-shaped, associated both-column, anterior column or wall with posterior hemitransverse, and posterior column with posterior wall

3. Please choose the right words to fill in the blanks to infer the mechanism of this fracture.

This acetabular fracture was caused by direct violent action on **(1)**, when the hip joint was **(2)** flexion and **(3)** abduction.

(1)	(2)	(3)
A. flexed knee	A. 0°	A. 0°
B. abdomen	B. 15°	B. 10°
C. lumbosacral area	C. 45°	C. 25°
D. greater trochanter	D. 90°	D. 60°

Questionnaire ID:

Acetabular fracture Learning Questionnaire(6)

Please choose your opinion on the following description.

(1) You enjoy this learning process.

strongly agree — agree — indifferent — disagree — strongly disagree

(2) This learning tool is simple and easy to use.

strongly agree — agree — indifferent — disagree — strongly disagree

(3) This learning tool can well present the acetabular fracture.

strongly agree — agree — indifferent — disagree — strongly disagree

(4) This learning tool is useful for learning acetabular fractures.

strongly agree — agree — indifferent — disagree — strongly disagree
